# Programmed death-1 expression on HIV-1-specific CD8^+^ T cells is shaped by epitope specificity, T-cell receptor clonotype usage and antigen load

**DOI:** 10.1097/QAD.0000000000000362

**Published:** 2015-08-27

**Authors:** Henrik N. Kløverpris, Reuben McGregor, James E. McLaren, Kristin Ladell, Anette Stryhn, Catherine Koofhethile, Jacqui Brener, Fabian Chen, Lynn Riddell, Luzzi Graziano, Paul Klenerman, Alasdair Leslie, Søren Buus, David A. Price, Philip Goulder

**Affiliations:** a Department of Paediatrics, University of Oxford, Peter Medawar Building, Oxford; b Institute of Infection and Immunity, Cardiff University School of Medicine, Heath Park, Cardiff, United Kingdom; c Department of International Health, Immunology and Microbiology, University of Copenhagen, Copenhagen N, Denmark; d Department of Sexual Health, Royal Berkshire Hospital, Reading; e Department of Genitourinary Medicine, Northamptonshire Healthcare National Health Service Trust, Northampton General Hospital, Cliftonville, Northampton; f Department of Sexual Health, Wycombe Hospital, High Wycombe, Buckinghamshire; g The Peter Medawar Building for Pathogen Research and NIHR Biomedical Research Centre, University of Oxford, South Parks Road, Oxford, United Kingdom; h KwaZulu-Natal Research Institute for Tuberculosis and HIV, K-RITH, Nelson R Mandela School of Medicine, University of KwaZulu-Natal, Durban, South Africa; i Human Immunology Section, Vaccine Research Center, National Institute of Allergy and Infectious Diseases, National Institutes of Health, Bethesda, Maryland, USA.; ∗ David A. Price and Philip Goulder contributed equally to this study.

**Keywords:** HIV, human leukocyte antigen class I tetramers, immune exhaustion, programmed death-1 expression

## Abstract

**Objectives:**

Although CD8^+^ T cells play a critical role in the control of HIV-1 infection, their antiviral efficacy can be limited by antigenic variation and immune exhaustion. The latter phenomenon is characterized by the upregulation of multiple inhibitory receptors, such as programmed death-1 (PD-1), CD244 and lymphocyte activation gene-3 (LAG-3), which modulate the functional capabilities of CD8^+^ T cells.

**Design and methods:**

Here, we used an array of different human leukocyte antigen (HLA)-B∗15 : 03 and HLA-B∗42 : 01 tetramers to characterize inhibitory receptor expression as a function of differentiation on HIV-1-specific CD8^+^ T-cell populations (*n* = 128) spanning 11 different epitope targets.

**Results:**

Expression levels of PD-1, but not CD244 or LAG-3, varied substantially across epitope specificities both within and between individuals. Differential expression of PD-1 on T-cell receptor (TCR) clonotypes within individual HIV-1-specific CD8^+^ T-cell populations was also apparent, independent of clonal dominance hierarchies. Positive correlations were detected between PD-1 expression and plasma viral load, which were reinforced by stratification for epitope sequence stability and dictated by effector memory CD8^+^ T cells.

**Conclusion:**

Collectively, these data suggest that PD-1 expression on HIV-1-specific CD8^+^ T cells tracks antigen load at the level of epitope specificity and TCR clonotype usage. These findings are important because they provide evidence that PD-1 expression levels are influenced by peptide/HLA class I antigen exposure.

## Introduction

Persistent viral infections necessitate lifelong host immunity. In this setting, efficacy is mediated predominantly by CD8^+^ T cells, which recognize virus-derived peptide epitopes presented on the surface of infected cells by human leukocyte antigen (HLA) class I molecules. Polymorphisms within the HLA class I locus contribute significantly to disease outcome in many such infections, including HIV-1, most likely via effects that intertwine with the quality of the cognate CD8^+^ T-cell response [1–3].

In addition to the requirement for durable protection and surveillance, there is a concomitant need to limit the pathology associated with continuous immune activation in the face of viral persistence [4]. The systematic upregulation of multiple inhibitory receptors on the surface of antigen-specific CD8^+^ T cells provides one such mechanism, which operates via the delivery of negative signals at various stages of the cellular differentiation programme [5,6]. This process is intimately linked with the phenomenon of ‘exhaustion,’ whereby effector T-cell functions are progressively lost according to a predictable hierarchy [7]. A finely tuned balance between the differentiation-linked acquisition and inhibitory receptor-mediated modulation of functional competence must therefore be achieved, either within a specialized phenotype [8] or within the heterogeneous pool of memory CD8^+^ T cells, to ensure an optimal outcome for the host.

Programmed death-1 (PD-1), a member of the CD28/CTLA-4 family, represents the prototype inhibitory receptor. The importance of PD-1 with respect to CD8^+^ T-cell exhaustion was first realized in the lymphocytic choriomeningitis virus (LCMV) model with the demonstration that antibody-mediated blockade reversed effector dysfunction and enhanced viral control [9]. Subsequent studies confirmed the human disease relevance of these findings, describing profound PD-1 upregulation on CD8^+^ T-cell populations specific for hepatitis C virus (HCV) [10,11], hepatitis B virus (HBV) [12,13] and HIV-1 [14–17]. In the latter case, PD-1 expression levels correlated with disease progression [14] and were shown to be reduced by either viral escape or treatment-induced suppression of antigen exposure. These observations strongly suggest that antigen-specific stimulation via the T-cell receptor (TCR) plays an important role in the regulation of PD-1 expression [14,16,18]. Moreover, treatment of simian immunodeficiency virus (SIV)-infected macaques with PD-1-blocking antibodies enhanced CD8^+^ T-cell immunity and prolonged survival [19].

Recently, it has been shown that PD-1 inhibits CD8^+^ T-cell function via upregulation of the basic leucine zipper transcription factor (BATF) [20]. It is also clear that other inhibitory receptors contribute to the functional impairment of CD8^+^ T cells in the setting of persistent viral infections, including CD160, CD244, lymphocyte activation gene-3 (LAG-3) and T-cell immunoglobulin and mucin domain-3 (Tim-3) [21–26]. Although these molecules offer novel opportunities for therapeutic intervention, a detailed understanding of the corresponding immunobiology will likely be required to inform rational progress in the clinic. In this light, it is established that inhibitory receptor expression profiles are tightly regulated at the transcriptional level [5,6]. Beyond a requirement for antigen exposure *per se*, however, the environmental cues associated with these transcriptional programmes remain less well defined.

In this study, we set out to determine the factors that govern inhibitory receptor expression on HIV-1-specific CD8^+^ T cells during the chronic phase of infection. Our analysis was restricted to two HLA class I molecules, HLA-B∗15 : 03 and HLA-B∗42 : 01, both of which occur at high frequencies in sub-Saharan Africa and present multiple different HIV-1-derived epitopes. This experimental design enabled controlled comparisons across epitope specificities within and between individuals. The importance of our approach is highlighted by observations of epitope-linked differences in HIV-1-specific CD8^+^ T-cell efficacy [1,27–30], which can further segregate at the level of individual TCR clonotypes [31,32].

## Methods

### Subjects

Individuals expressing either HLA-B∗15 : 03 (*n* = 15) or HLA-B∗42 : 01 (*n* = 17) were selected from a total cohort of 237 antiretroviral treatment-naïve participants with chronic HIV-1 infection [33,34]. The only additional criterion for selection was the availability of cryopreserved peripheral blood samples. All 32 participants were infected with HIV-1 clade C and harbored virus-specific CD8^+^ T-cell responses characterized previously by comprehensive interferon (IFN)γ ELISpot screening [28]. For the purposes of this study, 11 different CD8^+^ T-cell specificities were considered for detailed evaluation (Table S1). Informed consent was obtained from all participating individuals, and institutional review boards at the University of Oxford approved the study (E028/99). The use of material from human participants was conducted in accordance with the guidelines of the World Medical Association's Declaration of Helsinki (59th General Assembly).

### Human leukocyte antigen class I genotyping and viral load determination

Four-digit genotyping of HLA-A, HLA-B and HLA-C alleles was performed using Dynal REALTIME reverse sequence-specific oligonucleotide kits as described previously [28]. Viral loads were determined using the Roche Amplicor assay (version 1.5; Roche Molecular Diagnostics).

### Human leukocyte antigen class I tetramers

Biotinylated HLA-B∗42 : 01 monomers were generated according to standard protocols [35]. Tetramerization was performed by conjugation to extravidin-R-phycoerythrin (Sigma-Aldrich, St Louis, Missouri, USA). Tetrameric HLA-B∗15 : 03 complexes were generated as described previously [36]. The peptides used for HLA class I tetramer generation are shown in Table S1.

### Flow cytometry

A total of 128 HIV-1-specific CD8^+^ T-cell populations were studied. Frozen peripheral blood mononuclear cells (PBMCs) were thawed into Roswell Park Memorial Institute (RPMI) medium containing 20% fetal calf serum, rested for 1 h at 37°C in a 5% CO_2_ atmosphere, stained with the appropriate HLA class I tetramer for 30 min at room temperature, washed and then surface-stained with an anchor panel of monoclonal antibodies (mAbs) comprising αCD3 PacificOrange, αCD8 QD605, αCD14 PacificBlue and αCD19 PacificBlue (Life Technologies, Invitrogen). Dead cells were excluded from the analysis using LIVE/DEAD Fixable Violet (Life Technologies). Two distinct phenotypic panels were used for HLA-B∗15 : 03-restricted and HLA-B∗42 : 01-restricted responses, respectively: αCD45RA AlexaFluor700 (BD Biosciences, San Jose, California, USA), αCD57 FITC (BD Biosciences), αCD127 PE-Cy5 (eBioscience, San Diego, California, USA), αCCR7 PE-Cy7 (BD Biosciences) and αPD-1 APC (eBioscience); αCD45RA AlexaFluor700 (BD Biosciences), αCCR7 PE-Cy7 (BD Biosciences), αCD244 PE-Cy5 (BioLegend, San Diego, California, USA), αLAG-3 FITC (R&D Systems, Minneapolis, Minnesota, USA) and αPD-1 APC (eBioscience). In a subset of HLA-B∗42 : 01^+^ individuals, HIV-1-specific CD8^+^ T-cell populations were stained with PE-conjugated tetramers as described above, then surface-stained with αCD3 PacificOrange (Life Technologies), αCD8 V450 (BD Biosciences), αCD45RA AlexaFluor700 (BD Biosciences), αCCR7 PE-Cy7 (BD Biosciences), αCD244 PE-Cy5 (BioLegend), αPD-1 APC (eBioscience) and αTCRVβ mAbs conjugated to FITC (Beckman Coulter Inc, Miami, Florida, USA). Dead cells were excluded in the near-red spectrum (Life Technologies). In all cases, cells were stained with pretitrated mAbs for 30 min at room temperature, washed in PBS and fixed in 2% paraformaldehyde. Data were acquired within 12 h using an LSR II flow cytometer (BD Biosciences) and analyzed with FlowJo software version 8.8.6 (TreeStar Inc, Ashland, Oregon, USA), PESTLE version 1.6.2 and SPICE version 4.3 (Mario Roederer, National Institutes of Health, USA). Cells were hierarchically gated on singlets, lymphocytes and live CD3^+^ cells prior to Boolean analysis of tetramer-positive and tetramer-negative CD8^+^ cells, and further downstream gating on phenotypic markers as indicated. Median fluorescence intensity (MFI) values were calculated using FlowJo software version 8.8.6 (TreeStar Inc). All samples were run simultaneously to reduce assay variability. Fluorescence minus one (FMO) controls were used to set gates for markers with nondiscrete expression profiles.

### Analysis of T-cell receptor usage

Viable CD3^+^CD8^+^ HLA-B∗42 : 01 tetramer-positive cells were sorted at more than 98% purity using a modified FACSAria II flow cytometer (BD Biosciences) directly into 1.5-ml microfuge tubes (Sarstedt) containing 100 μl of RNAlater (Life Technologies). Molecular analysis of all expressed *TRB* gene rearrangements was subsequently conducted using an unbiased template-switch anchored reverse transcription PCR as described previously [37–39]. The international ImMunoGeneTics (IMGT) nomenclature is used throughout this manuscript [40].

### Quantification of functional sensitivity (EC_50_)

The peptide concentration required to elicit 50% of the maximum response magnitude [EC_50_ (μg/ml)] was determined by IFNγ ELISpot analysis [28]. Optimal peptides were used as stimulants and titrated across a concentration gradient of eight logs in 10-fold serial dilutions.

### Autologous proviral DNA sequencing

Genomic DNA was extracted from PBMCs and amplified by nested PCR using previously published primers [41,42]. The resultant PCR products were purified as described previously [43]. Sequencing was performed using the Big Dye Terminator v3.1 Cycle Sequencing Kit (Life Technologies) [44,45].

### Statistical analysis

The Mann–Whitney *U* test was used to compare median values with respect to the expression of phenotypic markers on bulk and tetramer-positive CD8^+^ T cells, both in terms of cell percentages and fluorescence intensities. The Holm–Sidak analysis of variance test was used for multiple comparisons across responses with respect to both parent gate percentage and MFI values. The Wilcoxon signed-rank test was used to compare median values with respect to differences between CD8^+^ T-cell memory populations. The Spearman rank test was used to determine correlations between cell percentages with respect to the parent gate and MFI values. Analyses were conducted using GraphPad Prism version 6.0 (GraphPad Software, La Jolla, California, USA). The Student *t* test was used to calculate differences between CD8^+^ T-cell populations specific for FL9-Vpr and other HIV-1-derived epitopes as determined by Boolean gating (SPICE version 4.3).

## Results

### Increased programmed death-1 and CD244 expression on HIV-1-specific CD8^+^ T cells

**Fig. 1:**
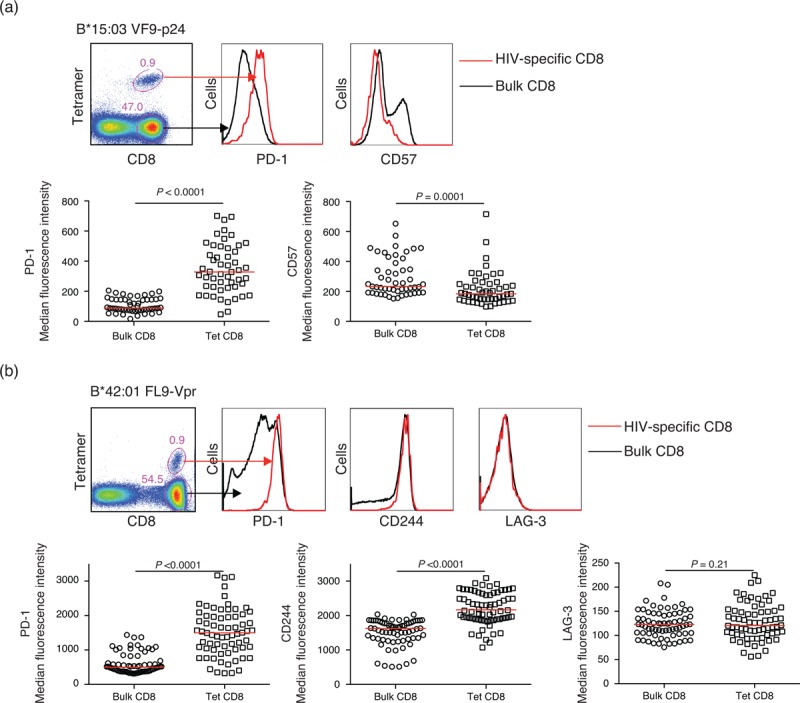
Increased programmed death-1 (PD-1) and CD244 expression on HIV-1-specific CD8^+^ T cells.

To investigate the expression of exhaustion markers on HIV-1-specific CD8^+^ T cells across multiple epitope targets with identical restriction elements, we used four HLA-B∗15 : 03 and seven HLA-B∗42 : 01 tetramers (Table S1) to stain PBMC samples directly *ex vivo* from individuals with chronic untreated HIV-1 clade C infection (*n* = 15 and *n* = 17, respectively). Surface expression of the differentiation marker CD57 and three inhibitory markers (PD-1, CD244 and LAG-3), previously shown to be upregulated during chronic viral infections [21,24,25], was determined by polychromatic flow cytometry. The vast majority of HIV-1-specific CD8^+^ T cells were found to reside in the PD-1^high^, CD244^high^ and CD57^low^ compartments (Fig. 1). Analysis of all HLA-B∗15 : 03-restricted (*n* = 52) and HLA-B∗42 : 01-restricted (*n* = 76) HIV-1-specific CD8^+^ T-cell populations revealed that both PD-1 and CD244 were upregulated compared with tetramer-negative bulk CD8^+^ T cells (*P* < 0.0001), whereas CD57 expression was decreased (*P* = 0.0001) (Fig. 1a and b). No differences in LAG-3 expression were detected between HIV-1-specific and bulk CD8^+^ T cells (Fig. 1b).

(a) PD-1 and CD57 expression on human leukocyte antigen (HLA)-B∗15 : 03 VF9-p24 tetramer-positive (red lines) and tetramer-negative bulk (black lines) CD8^+^ T cells (top). Median fluorescence intensity values for PD-1 and CD57 across all (*n* = 52) HLA-B∗15 : 03-restricted tetramer-positive compared to tetramer-negative bulk CD8^+^ T cells (bottom). (b) PD-1, CD244 and lymphocyte activation gene-3 (LAG-3) expression on HLA-B∗42 : 01 FL9-Vpr tetramer-positive (red lines) and tetramer-negative bulk (black lines) CD8^+^ T cells (top). Median fluorescence intensity values for PD-1, CD244 and LAG-3 across all (*n* = 71) HLA-B∗42 : 01-restricted tetramer-positive compared to tetramer-negative bulk CD8^+^ T cells (bottom). Statistical analyses were conducted using the Mann–Whitney *U* test.

### Differential epitope-linked expression of programmed death-1 on HIV-1-specific CD8^+^ T cells

Previous studies have compared the expression of negative regulatory molecules on HIV-1-specific CD8^+^ T cells to other persistent viral specificities, such as cytomegalovirus and Epstein-Barr virus (EBV) [25,38,46]. However, such comparisons ignore potential differences related to the targeted viral proteins or epitopes, even though fine specificity is linked to disparate CD8^+^ T-cell-mediated outcomes in HIV-1 infection [2]. To seek evidence of differential epitope-linked exhaustion, we first examined the expression of PD-1, CD57 and CD127 on CD8^+^ T-cell populations specific for distinct HIV-1-derived epitopes (*n* = 4) restricted by HLA-B∗15 : 03 (Table S1). Substantial differences were apparent across epitope specificities, most notably with respect to PD-1 expression (Fig. 2 a and b). In particular, CD8^+^ T-cell populations specific for VF9-p24, previously associated with effective immune control of HIV-1 replication [28], expressed high levels of PD-1 and low levels of CD127. The converse applied to CD8^+^ T-cell populations specific for FY10-Tat, which are not associated with protection [28]. These epitope-specific phenotypic differences were also apparent in terms of percentage expression frequencies (Fig. S1a and c). The expression of CD57 was more consistent across epitope specificities (Fig. 2 a and b, Fig S1b).

**Fig. 2:**
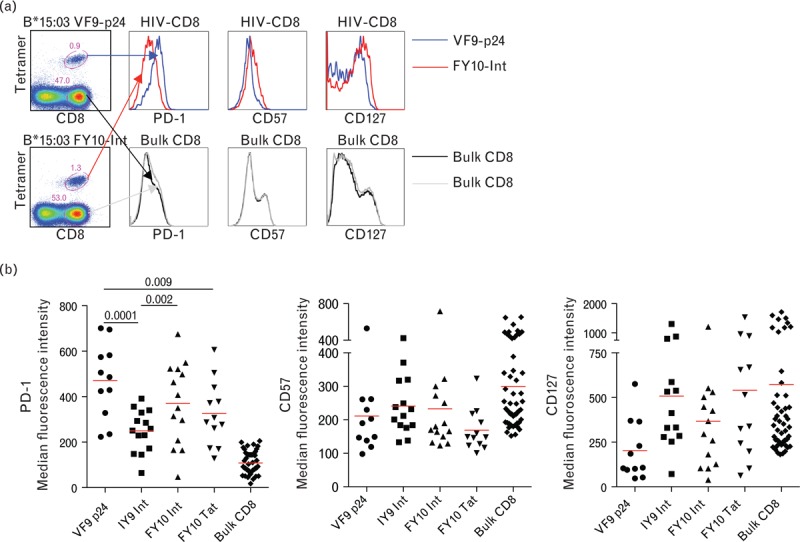
Differential epitope-linked expression of programmed death-1 (PD-1) on HIV-1-specific CD8^+^ T cells.

(a) PD-1, CD57 and CD127 expression on human leukocyte antigen (HLA)-B∗15 : 03 VF9-p24 tetramer-positive (blue lines) and HLA-B∗15 : 03 FY10-Int tetramer-positive (red lines) CD8^+^ T cells (top), with corresponding data for tetramer-negative bulk CD8^+^ T cells (bottom). (b) Median fluorescence intensity values for PD-1, CD57 and CD127 comparing each of the four different HLA-B∗15 : 03-restricted epitope-specific CD8^+^ T-cell populations and matched bulk CD8^+^ T cells (*n* = 52). (c) PD-1, CD244 and lymphocyte activation gene-3 (LAG-3) expression on HLA-B∗42 : 01 TL9-p24 tetramer-positive (blue lines) and HLA-B∗42 : 01 FL9-Vpr tetramer-positive (red lines) CD8^+^ T cells (top), with corresponding data for tetramer-negative bulk CD8^+^ T cells (bottom). (d) Median fluorescence intensity values for PD-1, CD244 and LAG-3 comparing each of the seven different HLA-B∗42 : 01-restricted epitope-specific CD8^+^ T-cell populations and matched bulk CD8^+^ T cells (*n* = 76). (e) Boolean gating of the seven different HLA-B∗42 : 01-restricted epitope-specific CD8^+^ T-cell populations for all permutations of PD-1, CD244 and LAG-3 expression (x-axis). Corresponding data were not available for HLA-B∗15 : 03-restricted epitope-specific CD8^+^ T-cell populations. Bars represent mean values of percentage tetramer-gated HIV-1-specific CD8^+^ T-cell frequencies (y-axis). Error bars represent standard error of the mean. ‘+’ indicates *P* < 0.05 compared with FL9-Vpr values (Student *t* test). Aggregated data are shown for 17 participants. Bulk CD8^+^ T cells represent tetramer-negative populations from HLA-B∗15 : 03^+^ and HLA-B∗42 : 01^+^ individuals. Adjusted *P* values (*P* < 0.05) for multiple comparisons in (b) and (d) were calculated using the Holm–Sidak analysis of variance test. Significant differences with respect to bulk CD8^+^ T cells are not shown.

**Fig. 2 (Continued):**
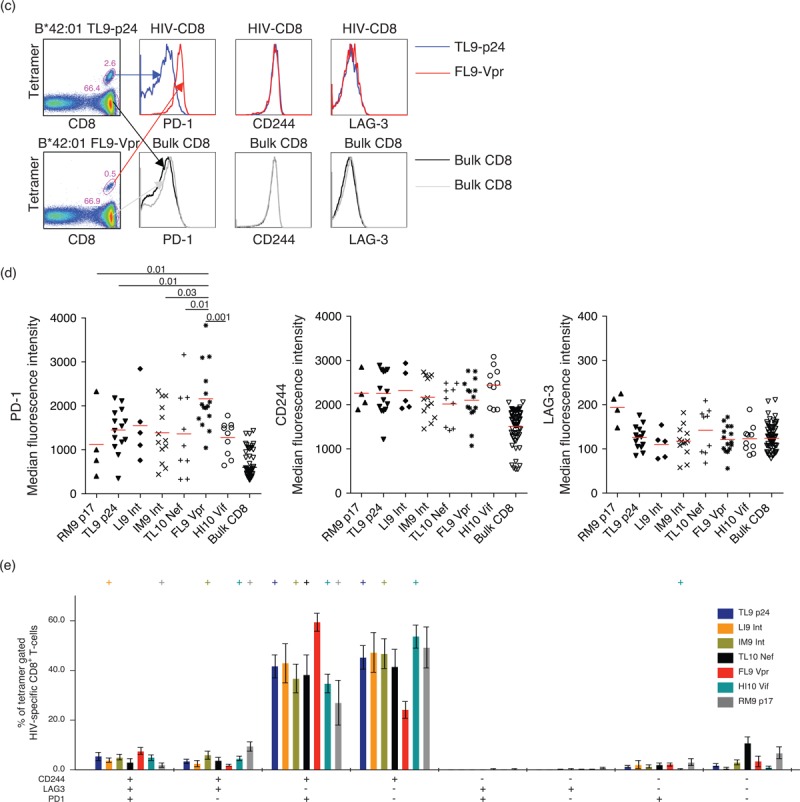
Differential epitope-linked expression of programmed death-1 (PD-1) on HIV-1-specific CD8^+^ T cells.

(a) PD-1, CD57 and CD127 expression on human leukocyte antigen (HLA)-B∗15 : 03 VF9-p24 tetramer-positive (blue lines) and HLA-B∗15 : 03 FY10-Int tetramer-positive (red lines) CD8^+^ T cells (top), with corresponding data for tetramer-negative bulk CD8^+^ T cells (bottom). (b) Median fluorescence intensity values for PD-1, CD57 and CD127 comparing each of the four different HLA-B∗15 : 03-restricted epitope-specific CD8^+^ T-cell populations and matched bulk CD8^+^ T cells (*n* = 52). (c) PD-1, CD244 and lymphocyte activation gene-3 (LAG-3) expression on HLA-B∗42 : 01 TL9-p24 tetramer-positive (blue lines) and HLA-B∗42 : 01 FL9-Vpr tetramer-positive (red lines) CD8^+^ T cells (top), with corresponding data for tetramer-negative bulk CD8^+^ T cells (bottom). (d) Median fluorescence intensity values for PD-1, CD244 and LAG-3 comparing each of the seven different HLA-B∗42 : 01-restricted epitope-specific CD8^+^ T-cell populations and matched bulk CD8^+^ T cells (*n* = 76). (e) Boolean gating of the seven different HLA-B∗42 : 01-restricted epitope-specific CD8^+^ T-cell populations for all permutations of PD-1, CD244 and LAG-3 expression (x-axis). Corresponding data were not available for HLA-B∗15 : 03-restricted epitope-specific CD8^+^ T-cell populations. Bars represent mean values of percentage tetramer-gated HIV-1-specific CD8^+^ T-cell frequencies (y-axis). Error bars represent standard error of the mean. ‘+’ indicates *P* < 0.05 compared with FL9-Vpr values (Student *t* test). Aggregated data are shown for 17 participants. Bulk CD8^+^ T cells represent tetramer-negative populations from HLA-B∗15 : 03^+^ and HLA-B∗42 : 01^+^ individuals. Adjusted *P* values (*P* < 0.05) for multiple comparisons in (b) and (d) were calculated using the Holm–Sidak analysis of variance test. Significant differences with respect to bulk CD8^+^ T cells are not shown.

Next, we studied an array of HIV-1-specific CD8^+^ T-cell populations restricted by HLA-B∗42 : 01, spanning seven different epitopes derived from six different viral proteins (Table S1). In these experiments, we focused on the inhibitory markers PD-1, CD244 and LAG-3. Again, PD-1 expression levels differed markedly between epitope specificities (Fig. 2 c and d). The highest levels were found on CD8^+^ T cells specific for FL9-Vpr (*P* < 0.01) (Fig. 2 d, Fig S1d). In comparison, expression levels of CD244 and LAG-3 were more consistent across epitope specificities (Fig. 2 d, Fig S1e and f;). Boolean gating of all seven different HLA-B∗42 : 01-restricted CD8^+^ T-cell populations confirmed the dominant role of PD-1 within the CD244^high^ subsets in terms of percentage expression frequency, especially with respect to the FL9-Vpr specificity (Fig. 2 e). Furthermore, tight correlations were observed between MFI values and percentage expression frequencies for all differentially expressed markers (Fig S2a–d).

These findings demonstrate that significant epitope-linked differences in PD-1 expression levels exist between HIV-1-specific CD8^+^ T-cell populations restricted by the same HLA class I molecule. The lack of contemporaneous phenotypic differences related to CD244 and LAG-3 suggests that distinct mechanisms may be driving the differential expression of these exhaustion markers.

A recent study found a positive correlation between TCR avidity and PD-1 expression [47], thereby providing a potential explanation for differential epitope-linked phenotypes. To test this possibility, we examined the functional sensitivity of HIV-1-specific CD8^+^ T-cell responses in IFNγ ELISpot assays conducted directly *ex vivo* using sample-matched PBMCs (Fig S3a–d). No correlations were detected between PD-1 expression and functional sensitivity for a total of 30 different CD8^+^ T-cell responses spanning 10 different HIV-1-derived epitopes (Fig S3e). Furthermore, there was no correlation between PD-1 expression and response magnitude (Fig S3f).

### Programmed death-1 expression on HIV-1-specific CD8^+^ T cells is a measure of antigen load

A previous study demonstrated that different epitope-specific CD8^+^ T-cell populations in the same individual expressed different levels of PD-1 [14]. However, the basis for such disparities was not fully elucidated. To pursue this line of investigation, we analyzed PD-1 expression at a given time point in an individual with CD8^+^ T-cell responses directed against five different epitopes derived from four different HIV-1 proteins restricted by two different HLA-B molecules (Fig. 3 a). The PD-1^high^ population varied from 86% (FL9-Vpr) to 37% (TL9-p24) of tetramer-positive CD8^+^ T cells. In contrast, CD244 expression exceeded 96% for all five CD8^+^ T-cell populations. Furthermore, we found distinct patterns of PD-1 expression across different HIV-1-derived epitope-specific CD8^+^ T-cell populations in participants with different levels of viremia (Fig. 3 b). These epitope-linked differences within and between samples applied to each of 33 participants analyzed in a similar manner (data not shown).

**Fig. 3:**
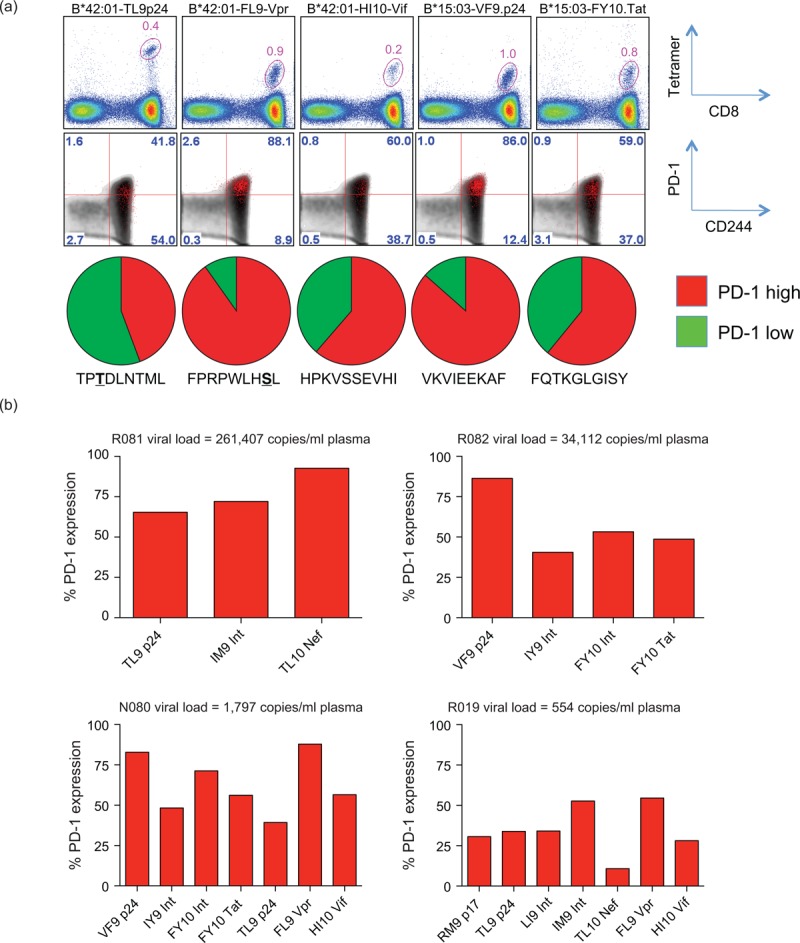
Programmed death-1 (PD-1) expression on HIV-1-specific CD8^+^ T cells is a measure of antigen load.

(a) PD-1 and CD244 expression on two different human leukocyte antigen (HLA)-B∗15 : 03-restricted and three different HLA-B∗42 : 01-restricted HIV-1-derived epitope-specific CD8^+^ T-cell populations present in the same sample from a single participant (N080). Tetramer-positive CD8^+^ T cells (red), gated as shown (top), are overlaid on bulk CD8^+^ cells (grey) in the cloud plots (middle). The pie charts show the proportion of PD-1^high^ (red) and PD-1^low^ (green) tetramer-positive CD8^+^ T cells within each antigen-specific population (bottom). Escape mutation residues in the corresponding cognate epitope sequences are underlined in bold. (b) Bars represent percentage frequencies for PD-1 expression on HIV-1-specific CD8^+^ T cells targeting different epitopes in four participants with different plasma viral loads. (c) Percentage frequencies for PD-1 expression on HIV-1-specific CD8^+^ T cells versus plasma viral loads (RNA copies/ml plasma) stratified for all epitope-specific responses (top left), responses targeting epitopes with low sequence variability (VF9-p24, TL9-p24 and TL10-Nef) (top right), and responses targeting epitopes with high sequence variability (IY9-Int, FY10-Int, FY10-Tat, RM9-p17, LI9-Int, IM9-Int, FL9-Vpr and HI10-Vif) (bottom left). The same correlation is also shown for tetramer-negative bulk CD8^+^ T cells (bottom right). Statistical values were calculated using the Spearman rank test.

**Fig. 3 (Continued):**
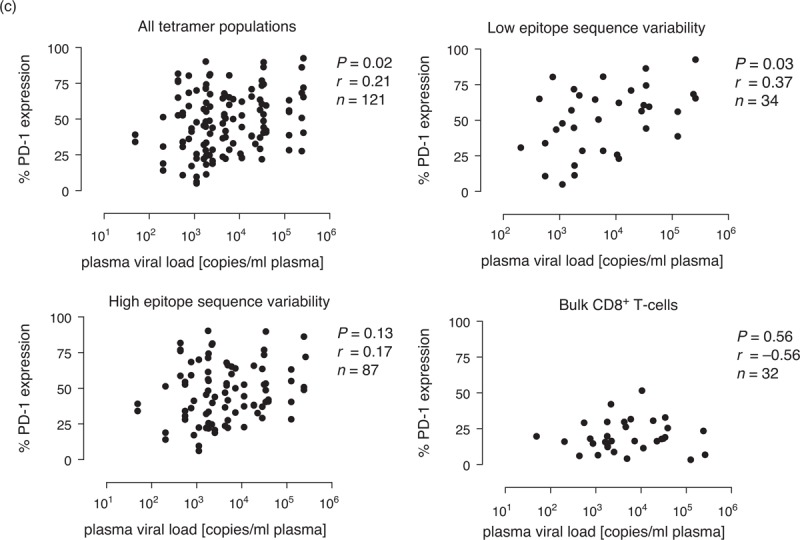
Programmed death-1 (PD-1) expression on HIV-1-specific CD8^+^ T cells is a measure of antigen load.

(a) PD-1 and CD244 expression on two different human leukocyte antigen (HLA)-B∗15 : 03-restricted and three different HLA-B∗42 : 01-restricted HIV-1-derived epitope-specific CD8^+^ T-cell populations present in the same sample from a single participant (N080). Tetramer-positive CD8^+^ T cells (red), gated as shown (top), are overlaid on bulk CD8^+^ cells (grey) in the cloud plots (middle). The pie charts show the proportion of PD-1^high^ (red) and PD-1^low^ (green) tetramer-positive CD8^+^ T cells within each antigen-specific population (bottom). Escape mutation residues in the corresponding cognate epitope sequences are underlined in bold. (b) Bars represent percentage frequencies for PD-1 expression on HIV-1-specific CD8^+^ T cells targeting different epitopes in four participants with different plasma viral loads. (c) Percentage frequencies for PD-1 expression on HIV-1-specific CD8^+^ T cells versus plasma viral loads (RNA copies/ml plasma) stratified for all epitope-specific responses (top left), responses targeting epitopes with low sequence variability (VF9-p24, TL9-p24 and TL10-Nef) (top right), and responses targeting epitopes with high sequence variability (IY9-Int, FY10-Int, FY10-Tat, RM9-p17, LI9-Int, IM9-Int, FL9-Vpr and HI10-Vif) (bottom left). The same correlation is also shown for tetramer-negative bulk CD8^+^ T cells (bottom right). Statistical values were calculated using the Spearman rank test.

Next, we extended this analysis to the entire dataset (Fig. 3 c). A weak correlation with viral load was detected for PD-1 expression levels across all HIV-1-specific CD8^+^ T-cell populations (*r* = 0.21, *P* = 0.02). To dissect this observation further, we stratified for viral escape mutations by separating epitopes with high (>40%) sequence variability (IY9-Int, FY10-Int, FY10-Tat, RM9-p17, LI9-Int, IM9-Int, FL9-Vpr and HI10-Vif) and low (<40%) sequence variability (VF9-p24, TL9-p24 and TL10-Nef) (Table S1). Strikingly, we found a stronger positive correlation between PD-1 expression and viral load for CD8^+^ T-cell populations targeting less variable epitopes (*r* = 0.37, *P* = 0.03). In contrast, no significant correlation was observed either for CD8^+^ T-cell populations targeting variable epitopes (*r* = 0.17, *P* = 0.13) or for bulk CD8^+^ T cells (*r* = −0.56, *P* = 0.56). Collectively, these data reveal a direct correlation between antigen load and PD-1 expression, which is lost when the virus generates escape mutations. This relationship suggests that PD-1 expression at the cellular level is driven directly by exposure to the cognate peptide/HLA complex.

### Different T-cell receptor clonotypes within individual HIV-1-specific CD8^+^ T-cell populations express different levels of programmed death-1

To dissect inhibitory receptor expression patterns in more detail, we examined the phenotypic profiles of individual clonotypes within HIV-1-specific CD8^+^ T-cell populations. Initially, we determined the clonotypic composition of HLA-B∗42 : 01 TL9-p24 tetramer-positive CD8^+^ T cells in a single participant (Fig. 4 a). The corresponding αTCRVβ mAbs were then used to identify clonotypic subsets within the epitope-specific CD8^+^ T-cell population, enabling their phenotypic characterization by flow cytometry (Fig. 4 b). Clear interclonotypic differences were apparent with respect to PD-1 expression; in contrast, no such differences were detected for CD244 (Fig. 4 c). Similar patterns were observed for a further five HIV-1-specific CD8^+^ T-cell populations targeting four distinct epitopes across five different participants (Fig. 4 d–f). However, there was no correlation between clonotypic dominance and PD-1 expression (Fig. 4 g) [47].

**Fig. 4:**
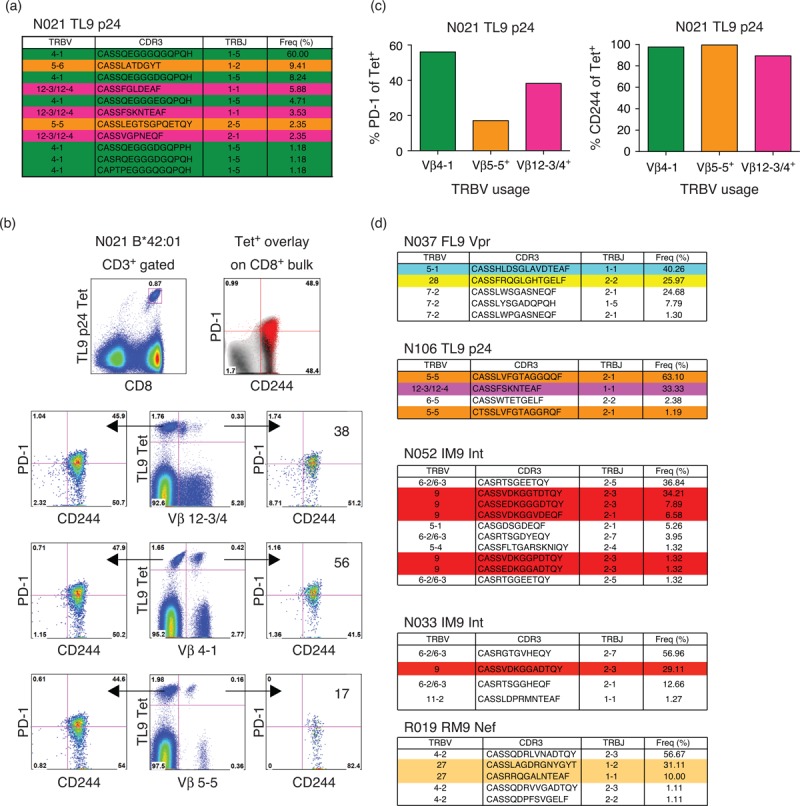
Different T-cell receptor (TCR) clonotypes within individual HIV-1-specific CD8^+^ T-cell populations express different levels of programmed death-1 (PD-1).

(a) Clonotypic composition of human leukocyte antigen (HLA)-B∗42 : 01 TL9-p24 tetramer-positive CD8^+^ T cells from participant N021 showing T-cell receptor beta variable (TRBV) usage, CDR3 amino acid sequence, T-cell receptor beta joining (TRBJ) usage and percentage frequency. Coloured highlights depict clonotypes with identical TRBV usage. (b) PD-1 and CD244 expression on HLA-B∗42 : 01 TL9-p24 tetramer-positive CD8^+^ T cells (red) overlaid on bulk CD8^+^ T cells (grey); data correspond to those shown in (a) from subject N021 (top). Combined tetramer and αTCRVβ staining, matched to the data shown in (a), depicting interclonotypic PD-1 and CD244 expression according to usage of Vβ12-3/4 (upper middle), Vβ4-1 (lower middle) and Vβ5-5 (bottom). (c) Percentage frequencies for PD-1 (left) and CD244 (right) expression on the TL9-p24 tetramer-positive Vβ^+^ cells shown in (b) colour-matched to the sequences shown in (a). (d) Clonotypic composition of HLA-B∗42 : 01 FL9-Vpr, TL9-p24, IM9-Int and RM9-Nef tetramer-positive CD8^+^ T cells from five different participants showing TRBV usage, CDR3 amino acid sequence, TRBJ usage and percentage frequency. Coloured highlights depict clonotypes with identical TRBV usage. (e and f) Percentage frequencies for PD-1 (e) and CD244 (f) expression on the tetramer-positive Vβ^+^ (colour-matched) and Vβ^−^ (black) cells shown in (d). (g) Comparison of PD-1 and CD244 expression frequencies on dominant, defined as the most frequent sequences identified in each epitope-specific CD8^+^ T-cell population depicted in (a) and (d), and subdominant clonotypes. Statistical analyses were conducted using the paired Student *t* test.

**Fig. 4 (Continued):**
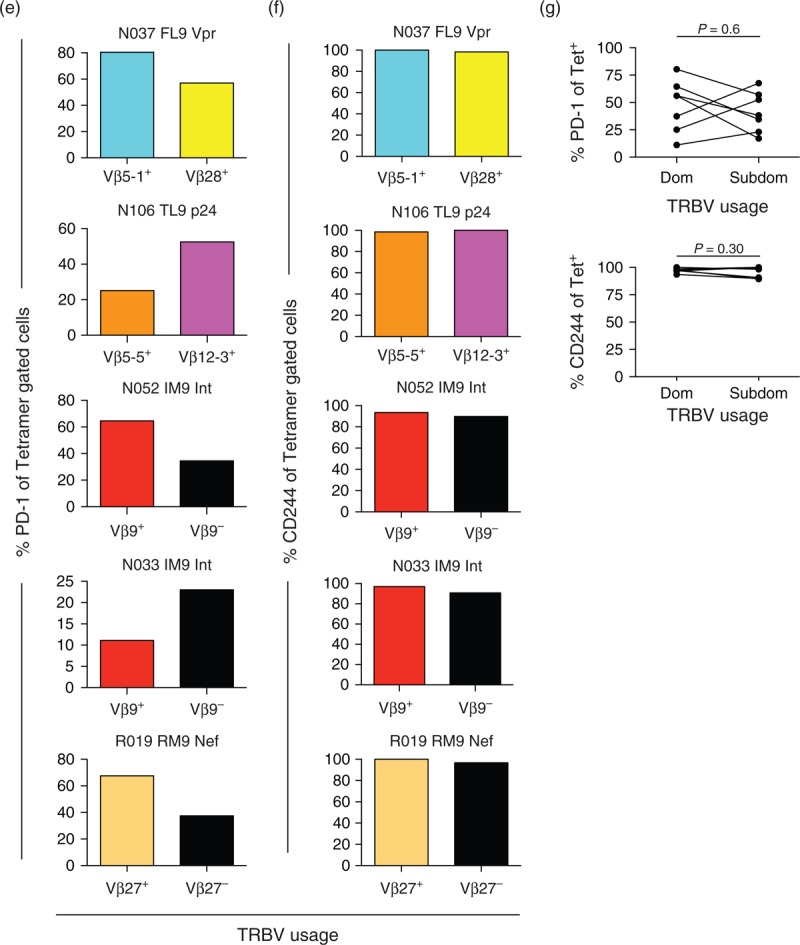
Different T-cell receptor (TCR) clonotypes within individual HIV-1-specific CD8^+^ T-cell populations express different levels of programmed death-1 (PD-1).

(a) Clonotypic composition of human leukocyte antigen (HLA)-B∗42 : 01 TL9-p24 tetramer-positive CD8^+^ T cells from participant N021 showing T-cell receptor beta variable (TRBV) usage, CDR3 amino acid sequence, T-cell receptor beta joining (TRBJ) usage and percentage frequency. Coloured highlights depict clonotypes with identical TRBV usage. (b) PD-1 and CD244 expression on HLA-B∗42 : 01 TL9-p24 tetramer-positive CD8^+^ T cells (red) overlaid on bulk CD8^+^ T cells (grey); data correspond to those shown in (a) from subject N021 (top). Combined tetramer and αTCRVβ staining, matched to the data shown in (a), depicting interclonotypic PD-1 and CD244 expression according to usage of Vβ12-3/4 (upper middle), Vβ4-1 (lower middle) and Vβ5-5 (bottom). (c) Percentage frequencies for PD-1 (left) and CD244 (right) expression on the TL9-p24 tetramer-positive Vβ^+^ cells shown in (b) colour-matched to the sequences shown in (a). (d) Clonotypic composition of HLA-B∗42 : 01 FL9-Vpr, TL9-p24, IM9-Int and RM9-Nef tetramer-positive CD8^+^ T cells from five different participants showing TRBV usage, CDR3 amino acid sequence, TRBJ usage and percentage frequency. Coloured highlights depict clonotypes with identical TRBV usage. (e and f) Percentage frequencies for PD-1 (e) and CD244 (f) expression on the tetramer-positive Vβ^+^ (colour-matched) and Vβ^−^ (black) cells shown in (d). (g) Comparison of PD-1 and CD244 expression frequencies on dominant, defined as the most frequent sequences identified in each epitope-specific CD8^+^ T-cell population depicted in (a) and (d), and subdominant clonotypes. Statistical analyses were conducted using the paired Student *t* test.

### Programmed death-1 expression on HIV-1-specific CD8^+^ T cells is driven by the effector memory population

It has been shown previously that PD-1 expression is enriched within the effector memory population of HIV-1-specific CD8^+^ T cells [46] and differentially expressed with CD57 [48]. To investigate differentiation-linked expression of PD-1 in our cohort, we pregated on PD-1^high^ and CD57^high^ populations, then compared tetramer-positive HIV-1-specific CD8^+^ T cells and tetramer-negative bulk CD8^+^ T cells with respect to memory phenotype (Fig. 5 a). Both PD-1^high^ and CD57^high^ populations were enriched in the T_EM_ compartment (Fig. 5 b and c), and differentially expressed on T_EM_ (CCR7^−^/CD45RA^−^) and terminally differentiated T_EMRA_ (CCR7^−^/CD45RA^+^) cells (Fig. 5 d and e). Furthermore, PD-1^high^ CD8^+^ T cells across all HIV-1-derived epitope specificities resided predominantly in the effector memory (CCR7^low^/CD45RA^low^) rather than the T_EMRA_ (CCR7^low^/CD45RA^high^) pool (Fig. 5 f and g), thereby supporting the hypothesis that PD-1 expression is driven by repetitive antigen exposure [49].

**Fig. 5:**
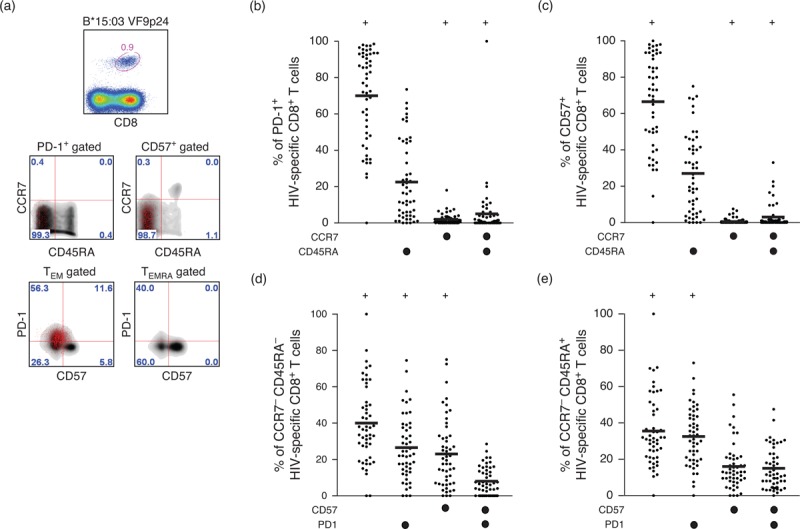
Programmed death-1 (PD-1) expression on HIV-1-specific CD8^+^ T cells is driven by the effecter memory population.

(a) Human leukocyte antigen (HLA)-B∗15 : 03 VF9-p24 tetramer-positive CD8^+^ T cells (top) pregated for PD-1 or CD57 expression and overlaid (red) on tetramer-negative bulk CD8^+^ T cells (grey) with respect to CCR7 and CD45RA expression (middle) or pregated for T_EM_ (CCR7^−^/CD45RA^−^) or T_EMRA_ (CCR7^−^/CD45RA^+^) phenotype and overlaid (red) on tetramer-negative bulk CD8^+^ T cells (grey) with respect to CD57 and PD-1 expression (bottom). (b and c) Expression of CCR7 and CD45RA (x-axis key) on a total of 52 different HLA-B∗15 : 03-restricted HIV-1-specific CD8^+^ T-cell populations from 15 participants pregated for PD-1 (b) or CD57 (c) positivity. (d and e) Expression of PD-1 and CD57 (x-axis key) on a total of 52 different HLA-B∗15 : 03-restricted HIV-1-specific CD8^+^ T-cell populations from 15 participants pregated for T_EM_ (d) or T_EMRA_ (e) phenotype. (f and g) Percentage frequencies for PD-1 expression on HLA-B∗42 : 01 (f) or HLA-B∗15 : 03 (g) tetramer-positive CD8^+^ T-cell populations within the T_EM_ (CCR7^−^/CD45RA^−^) or T_EMRA_ (CCR7^−^/CD45RA^+^) compartments. Horizontal bars depict mean values. In (b–c), ‘+’ indicates *P* < 0.05 compared with the CCR7^−^/CD45RA^+^ population. In (d–e), ‘+’ indicates *P* < 0.05 compared with the CD57^+^/PD-1^+^ population. In (f and g), ‘∗’ indicates *P* < 0.05 compared with FL9-Vpr values. Statistical analyses were conducted using the Student *t* test.

**Fig. 5 (Continued):**
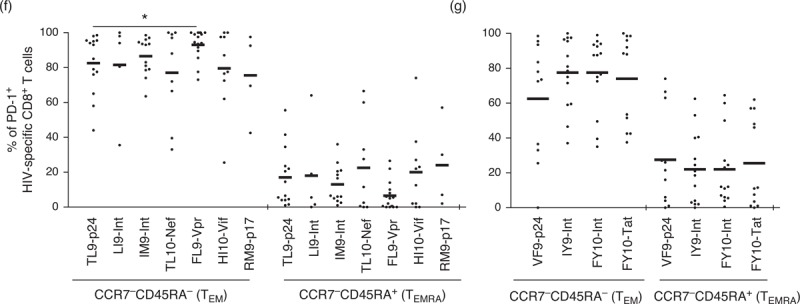
Programmed death-1 (PD-1) expression on HIV-1-specific CD8^+^ T cells is driven by the effecter memory population.

(a) Human leukocyte antigen (HLA)-B∗15 : 03 VF9-p24 tetramer-positive CD8^+^ T cells (top) pregated for PD-1 or CD57 expression and overlaid (red) on tetramer-negative bulk CD8^+^ T cells (grey) with respect to CCR7 and CD45RA expression (middle) or pregated for T_EM_ (CCR7^−^/CD45RA^−^) or T_EMRA_ (CCR7^−^/CD45RA^+^) phenotype and overlaid (red) on tetramer-negative bulk CD8^+^ T cells (grey) with respect to CD57 and PD-1 expression (bottom). (b and c) Expression of CCR7 and CD45RA (x-axis key) on a total of 52 different HLA-B∗15 : 03-restricted HIV-1-specific CD8^+^ T-cell populations from 15 participants pregated for PD-1 (b) or CD57 (c) positivity. (d and e) Expression of PD-1 and CD57 (x-axis key) on a total of 52 different HLA-B∗15 : 03-restricted HIV-1-specific CD8^+^ T-cell populations from 15 participants pregated for T_EM_ (d) or T_EMRA_ (e) phenotype. (f and g) Percentage frequencies for PD-1 expression on HLA-B∗42 : 01 (f) or HLA-B∗15 : 03 (g) tetramer-positive CD8^+^ T-cell populations within the T_EM_ (CCR7^−^/CD45RA^−^) or T_EMRA_ (CCR7^−^/CD45RA^+^) compartments. Horizontal bars depict mean values. In (b–c), ‘+’ indicates *P* < 0.05 compared with the CCR7^−^/CD45RA^+^ population. In (d–e), ‘+’ indicates *P* < 0.05 compared with the CD57^+^/PD-1^+^ population. In (f and g), ‘∗’ indicates *P* < 0.05 compared with FL9-Vpr values. Statistical analyses were conducted using the Student *t* test.

## Discussion

In this study, we conducted a detailed analysis of inhibitory receptor expression across a large number of different HIV-1-derived epitope-specific CD8^+^ T-cell populations restricted by either HLA-B∗15 : 03 or HLA-B∗42 : 01 to inform our understanding of the processes that regulate and potentially compromise antiviral immune responses. Major differences were observed for PD-1 expression levels across epitope specificities both within and between individuals. Differential interclonotypic expression of PD-1 was also apparent within individual HIV-1-specific CD8^+^ T-cell populations. Positive correlations were detected between PD-1 expression and plasma viral load, which were reinforced by stratification for epitope sequence stability and dictated by effector memory CD8^+^ T cells. Thus, PD-1 expression on HIV-1-specific CD8^+^ T cells is shaped by epitope specificity as a function of differentiation and driven by antigen load.

Our finding that PD-1 expression is increased on HIV-1-specific CD8^+^ T cells confirms previous studies [14,16] and concurs with similar observations in other persistent viral infections, including LCMV [9] and SIV [50]. However, it is established that multiple inhibitory receptors beyond PD-1 are involved in the negative regulation of CD8^+^ T-cell immunity [21]. Accordingly, we examined CD244 and LAG-3 expression in parallel. Consistent with a recent study [25], CD244 expression levels were increased on HIV-1-specific CD8^+^ T cells. In contrast, we found no evidence for elevated LAG-3 expression.

In addition to profound upregulation on HIV-1-specific CD8^+^ T cells as a whole, PD-1 expression also exhibited substantial differences between epitope specificities. The large number of HIV-1-derived epitopes restricted by only two HLA-B molecules examined in our study provides a distinct advantage over previous reports [16,17,47,51] in that it enables intraindividual comparisons between epitope specificities with an inbuilt control for the restriction element. In this setting, concomitant epitope-linked differences in CD244 and LAG-3 expression were not detected. These findings indicate that PD-1 expression is regulated more stringently in an antigen-dependent manner.

Previous reports have suggested that TCR avidity is linked to PD-1 expression as a function of signal strength [47]. In functional assays across a subset of matched samples, we found no evidence to support this hypothesis [52]. However, this finding is subject to one important caveat. Specifically, the measurement of functional sensitivity in this setting does not necessarily act as a reliable surrogate for TCR avidity because cytokine output cannot be assumed as a constant. Indeed, PD-1 expression preferentially inhibits poorly sensitive functions, such as IFNγ, thereby skewing functional assays toward an inverse relationship.

One important aspect of our study is the identification of multiple different HIV-1-derived epitope-specific CD8^+^ T-cell populations within individual samples, thereby providing intrinsic controls for interparticipant variables, such as plasma viral load. Accordingly, we could link differences in PD-1 expression to individual epitope-specific CD8^+^ T-cell populations in the absence of important confounders. The large variations in PD-1 expression between different epitope specificities were not limited to participants with high levels of viremia, but applied across the spectrum of plasma viral loads detected in the present cohort. This finding suggests that epitope-specific effects on PD-1 expression levels are paramount. Similar data have recently been reported for different EBV-specific CD8^+^ T-cell populations restricted by HLA-A∗02 : 01 [53].

Strikingly, PD-1 expression levels also differed between TCR clonotypes within HIV-1-specific CD8^+^ T-cell populations. However, we found no relationship with clonal dominance, in contrast to a previous report [47]. Nonetheless, this observation is consistent with the lack of correlation between response magnitude and PD-1 expression in our cohort. A recent study demonstrated that PD-1 expression on CD8^+^ T cells is maintained by a mechanism of high production and high clearance [54]. Accordingly, if high avidity clonotypes preferentially acquire PD-1 expression, they may fail to dominate despite the operation of avidity-based selection [39]. These considerations add another layer of complexity to the array of forces that govern the clonotypic architecture of antigen-specific CD8^+^ T-cell populations. It remains to be determined whether interclonotypic differences in PD-1 expression are linked to differential antiviral activity [31,32,55,56].

The correlation between plasma viral load and PD-1 expression across all HIV-1-specific CD8^+^ T-cell populations is consistent with previous studies [14,17,25]. Moreover, this correlation was strengthened by stratification for targeted epitopes that undergo mutation less frequently, whereas no such association was detected for CD8^+^ T-cell populations directed against more variable epitopes. These findings suggest that PD-1 expression is regulated in an antigen-dependent manner, although the effect of viral load in this regard may still be indirect. Longitudinal studies demonstrating reduced PD-1 expression after mutational escape [18] and treatment-induced suppression of viral antigen load [25,51], as well as increased PD-1 expression after treatment interruption [52], support this interpretation. Nonetheless, multiple host-specific and virus-specific factors undoubtedly affect epitope processing and presentation [57], confounding any simplistic relationships between the parameters measured in this study. The previously reported inverse correlation between plasma viral load and PD-1 expression on HIV-1-specific CD8^+^ T cells during acute infection likely represents a separate scenario occurring before the onset of functional exhaustion [58], although the PD-1 promoter remains unmethylated and active throughout the course of infection [59].

Collectively, our data show that PD-1 expression on HIV-1-specific CD8^+^ T cells is tightly linked to epitope specificity and TCR clonotype usage regardless of plasma viral load during the chronic phase of infection. The observation that PD-1 levels are most strongly correlated with antigenemia stratified for epitope sequence stability suggests that PD-1 tracks peptide/HLA complexes visible to the cognate TCR. Accordingly, PD-1 expression on the mobilized CD8^+^ T-cell population may serve as a surrogate marker for epitope density on the surface of infected target cells.

## Acknowledgements

This work was supported by the Wellcome Trust (D.P. and P.G.) and the National Institutes of Health (grant #R01 AI46995). H.K. is funded by the Danish Agency for Science, Technology and Innovation (grant #12-132295). D.P. is a Wellcome Trust Senior Investigator.

H.K. designed the study, performed the experiments, and wrote the paper; R.M., J.M., K.L. and J.B. performed experiments; A.S. and C.K. generated HLA class I tetramers; F.C., L.R. and L.G. provided clinical specimens; P.K. and A.L. contributed to analysis and design; S.B. contributed to reagent provision; D.P. and P.G. contributed financial and intellectual input, and revised the manuscript.

### Conflicts of interest

All authors declare that no competing interests exist.

## Supplementary Material

Supplemental Digital Content
