# Supplementary material for: Programmed death-1 expression on HIV-1-specific CD8+ T cells is shaped by epitope specificity, T-cell receptor clonotype usage and antigen load
Source: AIDS. 2015 Aug 27;28(14):2007–21. doi: 10.1097/QAD.0000000000000362 (PMC4166042; doi:10.1097/QAD.0000000000000362)
Supplement: Supplemental Digital Content [file aids-28-2007-s001.pdf]

SUPPLEMENTARY FIGURE LEGENDS

Figure S1. Percentage expression of inhibitory receptors on HIV-1-specific CD8$^+$ T-cells. (a-c) Percentage frequencies for expression of PD-1 **(a)**, CD57 **(b)** and CD127 **(c)** on HLA-B*15:03 VF9-p24, IY9-Int, FY10-Int and FY10-Tat tetramer-positive CD8$^+$ T-cells. **(d-f)** Percentage frequencies for expression of PD-1 **(d)**, CD244 **(e)** and LAG-3 **(f)** on HLA-B*42:01 RM9-p17, TL9-p24, LI9-Int, IM9-Int, TL10-Nef, FL9-Vpr and HI10-Vif tetramer-positive CD8$^+$ T-cells. Tetramer-negative bulk CD8$^+$ T-cells are shown for comparison in each case. Adjusted P-values (P<0.05) for multiple comparison in **(b)** and **(d)** were calculated using the Holm-Sidak ANOVA test.

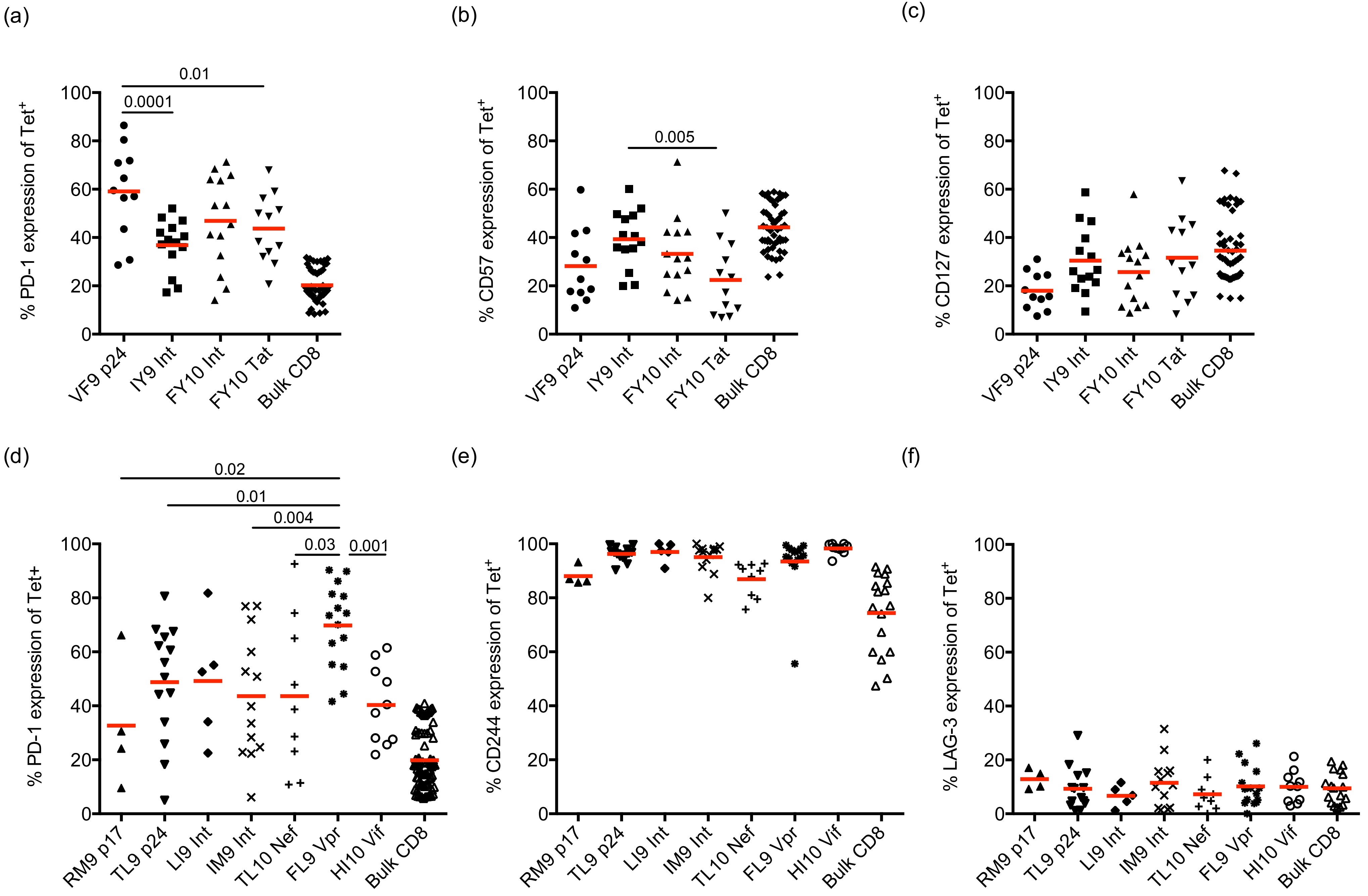

(a)
(b)
(c)
(d)
(e)
(f)
% PD-1 expression of Tet+
% CD57 expression of Tet+
% CD127 expression of Tet+
% PD-1 expression of Tet+
% CD244 expression of Tet+
% LAG-3 expression of Tet+
0.01
0.0001
0.005
0.02
0.01
0.004
0.03
0.001
VF9 p24
IY9 Int
FY10 Int
FY10 Tat
Bulk CD8
RM9 p17
TL9 p24
LI9 Int
IM9 Int
TL10 Nef
FL9 Vpr
HI10 Vif

Figure S2. Correlation between percentage frequency and median fluorescence intensity values for PD-1 expression on HIV-1-specific CD8^{+} T-cells. Median fluorescence intensity (MFI) versus percentage frequency for PD-1 **(a)**, CD57 **(c)** and CD127 **(d)** expression on HLA-B*15:03-restricted HIV-1-specific CD8^{+} T-cells and PD-1 **(b)** expression on HLA-B*42:01-restricted HIV-1-specific CD8^{+} T-cells. Antigen specificity was identified by tetramer staining. Statistical values were calculated using the Spearman rank test.

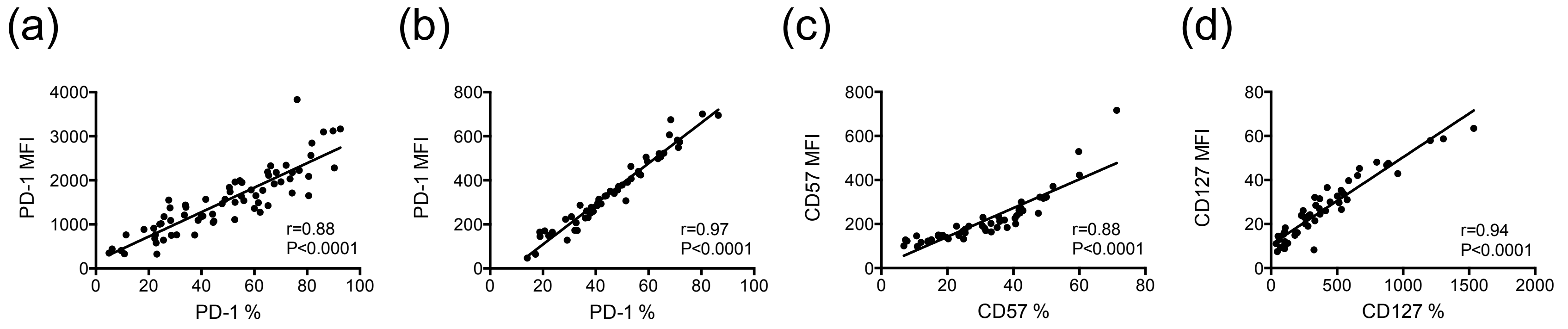

(a)
PD-1 MFI
PD-1 %
r=0.88
P<0.0001
(b)
PD-1 MFI
PD-1 %
r=0.97
P<0.0001
(c)
CD57 MFI
CD57 %
r=0.88
P<0.0001
(d)
CD127 MFI
CD127 %
r=0.94
P<0.0001

Figure S3. PD-1 expression on HIV-1-specific $CD8^+$ T-cells is not clearly related to functional sensitivity or response magnitude. (a&c) Dose-response titrations of optimal peptides corresponding to each of the four HLA-B*15:03-restricted epitopes (VF9-p24, IY9-Int, FY10-Int and FY10-Tat) **(a)** or five HLA-B*42:01-restricted epitopes (RM9-p17, TL9-p24, IM9-Int, FL9-Vpr and HI10-Vif **(c)** in IFNγ ELISpot assays using PBMCs from subjects with known HLA-B*15:03-restricted (R082) or HLA-B*42:01-restricted (R014) HIV-1-specific $CD8^+$ T-cell responses. **(b&d)** Functional sensitivity (EC50) values for HLA-B*15:03-restricted (n=16) **(b)** and HLA-B*42:01-restricted (n=14) **(d)** HIV-1-specific $CD8^+$ T-cell responses. Horizontal bars represent median values. **(e)** Functional sensitivity versus percent PD-1 expression on tetramer-positive $CD8^+$ T-cell populations (n=30) for responses shown in **(b)** and **(d)**. **(f)** Response magnitude (% tetramer-positive $CD3^+$ cells) versus percent PD-1 expression on tetramer-positive $CD8^+$ T-cell populations (n=127). In **(b)**, P-values were calculated using the Mann-Whitney U-test. In **(e)** and **(f)**, statistical values were calculated using the Spearman rank test.

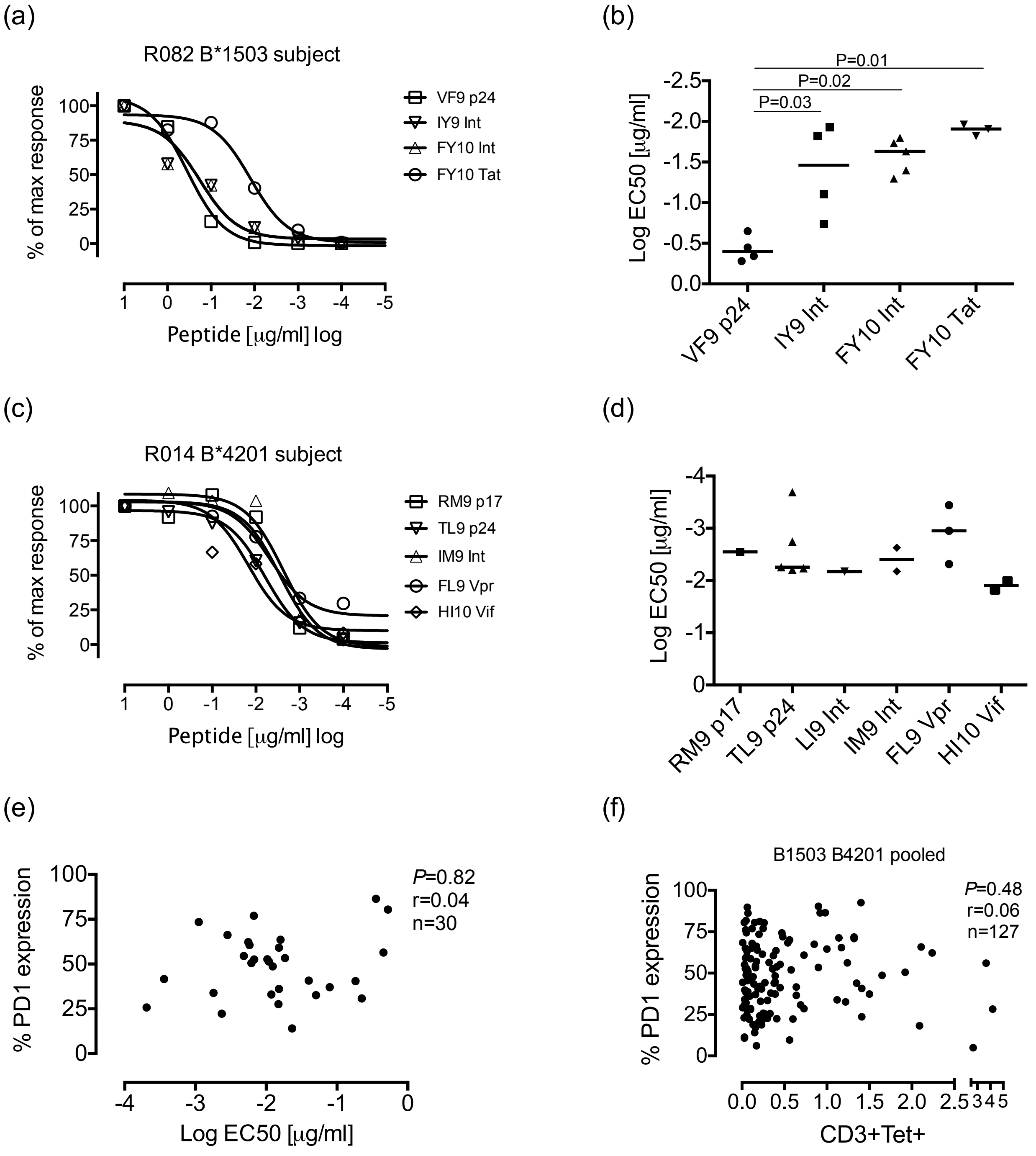

(a)
R082 B*1503 subject
% of max response
Peptide [μg/ml] log
VF9 p24
IY9 Int
FY10 Int
FY10 Tat
(b)
Log EC50 [μg/ml]
P=0.01
P=0.02
P=0.03
VF9 p24
IY9 Int
FY10 Int
FY10 Tat
(c)
R014 B*4201 subject
% of max response
Peptide [μg/ml] log
RM9 p17
TL9 p24
IM9 Int
FL9 Vpr
HI10 Vif
(d)
Log EC50 [μg/ml]
RM9 p17
TL9 p24
LI9 Int
IM9 Int
FL9 Vpr
HI10 Vif
(e)
% PD1 expression
Log EC50 [μg/ml]
P=0.82
r=0.04
n=30
(f)
B1503 B4201 pooled
% PD1 expression
CD3+Tet+
P=0.48
r=0.06
n=127

Table S1. Description of HLA class I tetramers used in the study

Tetramer HLA	Epitope Sequence	HXB2 location	HIV-1 protein	No. of responding subjects[a]	Median *ex vivo* frequency[b]
B*15:03 VF9	VKVIEEKAF	Gag156-164	p24	11	0.40
B*15:03 IY9	IQQEFGIPY	Pol850-858	Int	14	0.59
B*15:03 FY10	FKRKGGIGGY	Pol900-909	Int	15	0.83
B*15:03 FY10	FQTKGLGISY	Tat67-76	Tat	12	0.14
B*42:01 RM9	RPGGKKHYM	Gag22-30	p17	4	0.35
B*42:01 TL9	TPQDLNTML	Gag180-188	p24	15	1.50
B*42:01 LI9	LPPIVAKEI	Pol743-751	RT	5	0.09
B*42:01 IM9	IIKDYGKQM	Pol982-990	Int	13	0.24
B*42:01 TL10	TPGPGVRYPL	Nef128-137	Nef	9	0.09
B*42:01 FL9	FPRPWLHGL	Vpr34-42	Vpr	15	0.38
B*42:01 HI10	HPKVSSEVHI	Vif48-57	Vif	10	0.25

[a] Of a total of n=15 HLA-B*15:03^{+} and n=17 HLA-B*42:01^{+} subjects

[b]Percentage of CD3^{+}CD8^{+} gated tetramer-positive cells
